# The effect of thermal treatment on ac/dc conductivity and current fluctuations of PVDF/NMP/[EMIM][TFSI] solid polymer electrolyte

**DOI:** 10.1038/s41598-020-78363-6

**Published:** 2020-12-03

**Authors:** Petr Sedlak, Adam Gajdos, Robert Macku, Jiri Majzner, Vladimir Holcman, Vlasta Sedlakova, Petr Kubersky

**Affiliations:** 1 Faculty of Electrical Engineering and Communications, Brno University of Technology, Technická 10, Brno, 616 00 Czech Republic; 2 Faculty of Electrical Engineering, Regional Innovation Centre for Electric Engineering, University of West Bohemia, Univerzitni 8, Plzen, 301 00 Czech Republic

**Keywords:** Electronic properties and materials, Surfaces, interfaces and thin films, Sensors and biosensors, Chemical physics

## Abstract

The experimental study deals with the investigation of the effect of diverse crystallinity of imidazolium ionic-liquid-based SPE on conductivity and current fluctuations. The experimental study was carried out on samples consisting of [EMIM][TFSI] as ionic liquid, PVDF as a polymer matrix and NMP as a solvent. After the deposition, the particular sample was kept at an appropriate temperature for a specific time in order to achieve different crystalline forms of the polymer in the solvent, since the solvent evaporation rate controls crystallization. The ac/dc conductivities of SPEs were investigated across a range of temperatures using broadband dielectric spectroscopy in terms of electrical conductivity. In SPE samples of the higher solvent evaporation rate, the real parts of conductivity spectra exhibit a sharper transition during sample cooling and an increase of overall conductivity, which is implied by a growing fraction of the amorphous phase in the polymer matrix in which the ionic liquid is immobilized. The conductivity master curves illustrate that the changing of SPEs morphology is reflected in the low frequency regions governed by the electrode polarization effect. The dc conductivity of SPEs exhibits Vogel–Fulcher–Tammann temperature dependence and increases with the intensity of thermal treatment. Spectral densities of current fluctuations showed that flicker noise, thermal noise and shot noise seems to be major noise sources in all samples. The increase of electrolyte conductivity causes a decrease in bulk resistance and partially a decrease in charge transfer resistance, while also resulting in an increase in shot noise. However, the change of electrode material results in a more significant change of spectral density of current fluctuations than the modification of the preparation condition of the solid polymer electrolyte. Thus, the contact noise is considered to contribute to overall current fluctuations across the samples.

## Introduction

Solid polymer electrolytes (SPEs) are gaining attention and importance due to the growing scope of their application as electrolytes for batteries, super capacitors, fuel cells, sensors and other electrochemical devices^1–5^. SPEs offer several advantages over liquid electrolytes, such as good mechanical properties, ease of fabrication as flexible thin films in desirable size and the ability to form good electrode–electrolyte contact^1,2^. Thus, SPEs are suitable materials for printed and/or flexible electronic devices, such as batteries^6^, supercapacitors^7^, sensors^8–11^, etc. Nevertheless, printed SPE-based gas sensors may possess poor stability, low selectivity, complex sensitivity mechanisms, and short lifetimes^12^. Therefore, the development of a SPE, which offers improvements such as better stability, simple reaction mechanism, and longer life time, is an important motivation for current and future researches^13^.

SPEs are generally described as solid or gel ion-conducting membranes consisting of a salt dispersed in a polymer matrix forming a ionically conducting solid solution^14^. The most commonly studied polymer host for solid polymer electrolyte is polyethylene oxide (PEO) due to its applicability in solid lithium–polymer battery technology^4,15^ since PEO offers high power of solvation for lithium salts and good compatibility with electrodes^14^. However, PEO-based solid polymer electrolytes exhibit low ionic conductivity at ambient temperature^1,4,14^. Polyvinylidene fluoride (PVDF), another ideal candidate as polymer host for SPEs, has attracted researchers not only in the field of electrochemical devices, due to its chemical, thermal and electrical stability, as well as unique piezoelectric and pyroelectric properties^5,16,17^. Due to the linear structure and the mutual repulsion of fluorine atoms, PVDF could be of several kinds of crystal phase types, from which the α, β, and γ phases can exist in the PVDF matrix steadily^18^. The resulting crystalline phase depends on the crystallization rate^19^, which in turn is determined by the evaporation rate of the solvent. PVDF-based SPEs which consist of both crystalline and amorphous phases showed high dielectric constant that facilitates high charge dissociation and supports high concentration of charge carriers^18^, i.e. PVDF-based polymer electrolytes exhibited the highest conductivity at room temperature^20^.

There are many existing methods of modifying the conductivity of the polymer electrolyte, such as blending, copolymerization, cross-linking and the addition of additives^1,2,4,21–24^. The blending and adding nanofillers (such as carbon powder, nanowires, SWCNT, etc.) represent probably the most simple and effective ways to improve the mechanical and electrochemical properties^4^. However, the dispersion of these nanofillers could still poses significant challenges in applications^25^.

On the other hand, the suitable candidates for blending are room temperature ionic liquids (ILs) which offer low melting points, negligible vapor pressure, high chemical and thermal stabilities, high ionic conductivity, and a broad electrochemical potential window^1,3,20,25^. A benefit of ILs-based SPEs resides in the fact that ionic liquids allow almost unlimited structural variations, and it is therefore possible to tailor their electrical and mechanical properties according to functional requirements^2,20^. For example, Correia et al.^26,27^ showed that IL with different anions and cations, when incorporated into a PVDF matrix, will affect the crystallinity of PVDF in the composite as well as the mechanical properties, due to the interaction of the positive and negative polymer chains of PVDF with the anion and cation of IL, respectively. Beside the acting as a plasticizing agent, ILs also play the role of the supplier of ionic charge carrier to significantly change polymer dielectric behavior^28,29^. These effects on crystallization and conductivity are related to amount of IL in the polymer electrolyte^25,30–32^.

Thermal treatment is another way to change the electromechanical properties of PVDF electrolytes^16^. In addition to the content of IL, thermal annealing can also increase the polar phase and decrease the degree of crystallization for PVDF composites. Xu et al.^18^ report that the change of charge carrier movement mechanisms results in an increase in ion mobility induced by the polymer chain segmental motion and polar phase crystals. Dong et al.^29^ demonstrated on PVDF/[BMIM][TFSI] mixtures (dimethyl formamide was used as solvent) that the solvent evaporation temperature as well as concentration of ionic liquid had a significant influence on the morphology, degree of crystallinity, mechanical properties, and ionic conductivity of the prepared polymer electrolytes.

To the best of our knowledge, this work is the first attempt which describes the effect of thermal treatment conditions on ac/dc conductivity and also current fluctuations of PVDF/IL-based solid polymer electrolyte. The current fluctuations represent macroscopically a number of physical and chemical stochastic processes taking place in charge transport across the electrochemical device. Researchers use fluctuation analysis to extract more information about these processes^33–37^. In another words, noise spectroscopy gives integral information about a whole state of the electrochemical device and may inform about issues in a design of device, a quality of electrical contacts, long-term stability, degradation of electrochemical interface. Thus, noise measurement is used in corrosion rate evaluation, aging characterization of fuel cells or supercapacitors, enhancing the response of chemical sensors, etc.

## Results and discussion

Gregorio and Borges^19^ reported that solvent evaporation temperature has a strong impact on the type of the crystalline phase of the polymer (PVDF) in the solvent (NMP) and found a relationship between the porosity of the prepared layers and the crystallization temperature. Thermogravimetric analyses provided by Nespurek et al.^38^ demonstrated that thermal stability of PVDF and the IL [EMIM][TFSI] and the relatively low boiling point of NMP result in two well-separated processes (110 °C and 425 °C) in the SPE mixture. The influence of the first process, i.e. thermal treatment conditions, impacts the morphology and conductivity of SPE. To begin with, the change of morphology is briefly demonstrated, the electrical conductivity of ionic liquid is described and the impact of treatment conditions on electrical conductivity of SPE, as well as on current fluctuation, is discussed.

**Figure 1:**
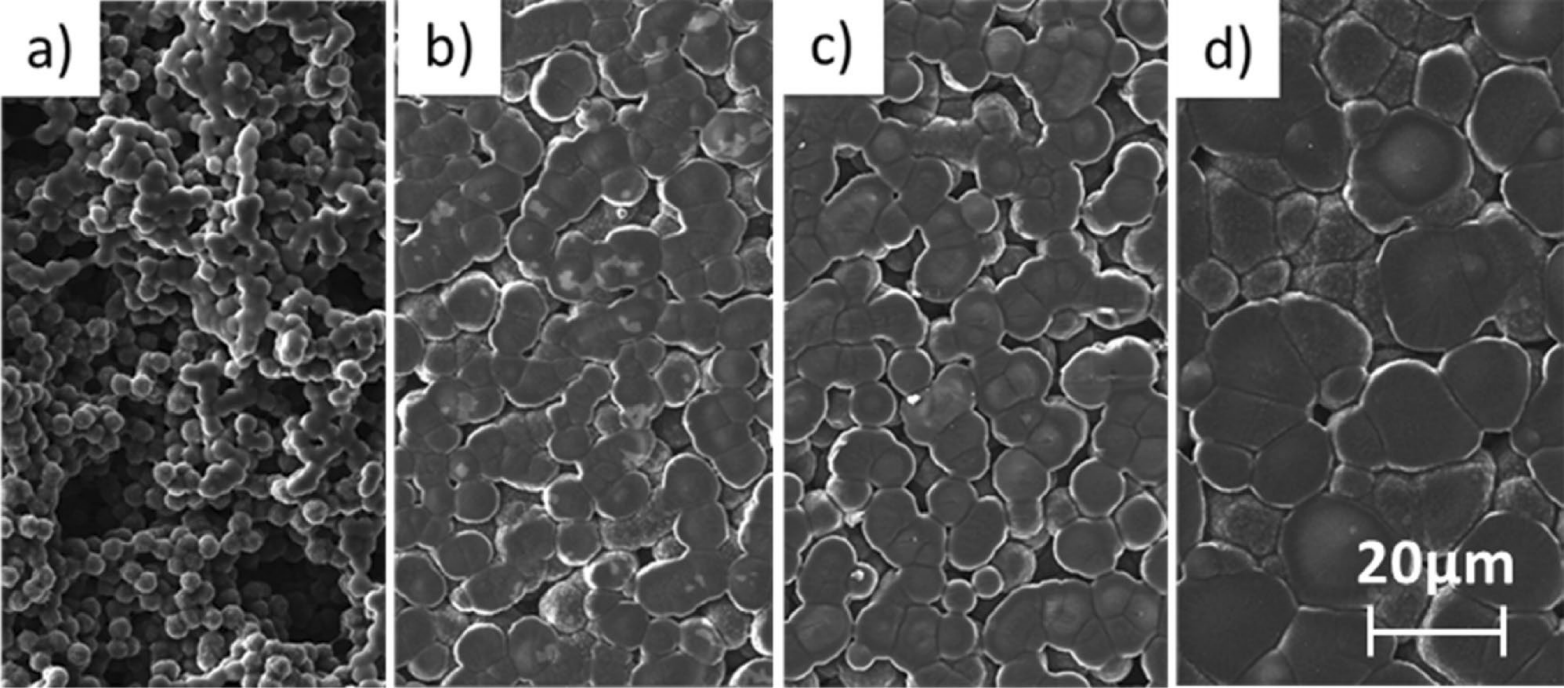
Morphology of the SPE based on imidazolium ionic liquid after the different preparation conditions: (**a**) 90 s 80 °C, (**b**) 90 s 120 °C, (**c**) 210 s 120 °C, (**d**) 600 s 160 °C.

Figure 1 illustrates the morphology of the SPEs based on imidazolium ionic liquid after different preparation conditions at the field of view of 100 μm in the middle of the SPE area. The surfaces consist of very small spherical SPE objects whose diameter increases with treatment temperature from ~ 3.6 up to ~ 14.82 μm; thus, the lower value of this temperature results in higher porosity of the prepared SPE. It needs to be noted that the diameter of spherical SPE objects differs in the middle and at the edges of the prepared layer on the top side, while the diameters measured on the bottom-side are similar to the values in the middle on the top side.

### Electrical conductivity of ionic liquid

**Figure 2:**
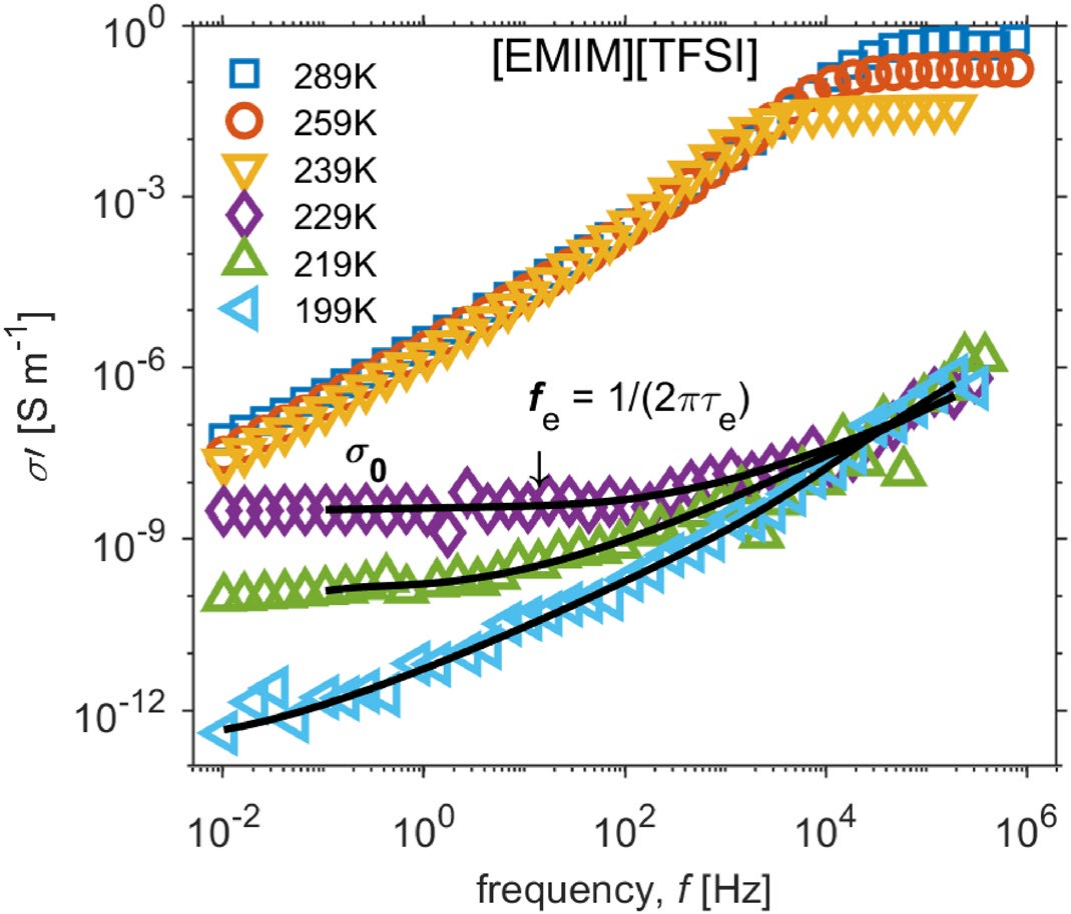
Dielectric relaxation spectra of [EMIM][TFSI] presented in the conductivity representation as function of frequency for selected temperatures at cooling. The black lines represent the fits on experimental data at particular temperatures (229 K, 219 K and 199 K ). The fits are made by combining both the Dyre’s random free energy barrier model and ‘relaxation-like’ process by Havriliak–Negami function.

The conductivity was investigated on the basis of wide band dielectric spectroscopy. Typical spectra of [EMIM][TFSI] between 0.01 Hz and 1 MHz are shown in Fig. 2, where real parts of electrical conductivity (σ′) are plotted for selected temperatures.

**Figure 3:**
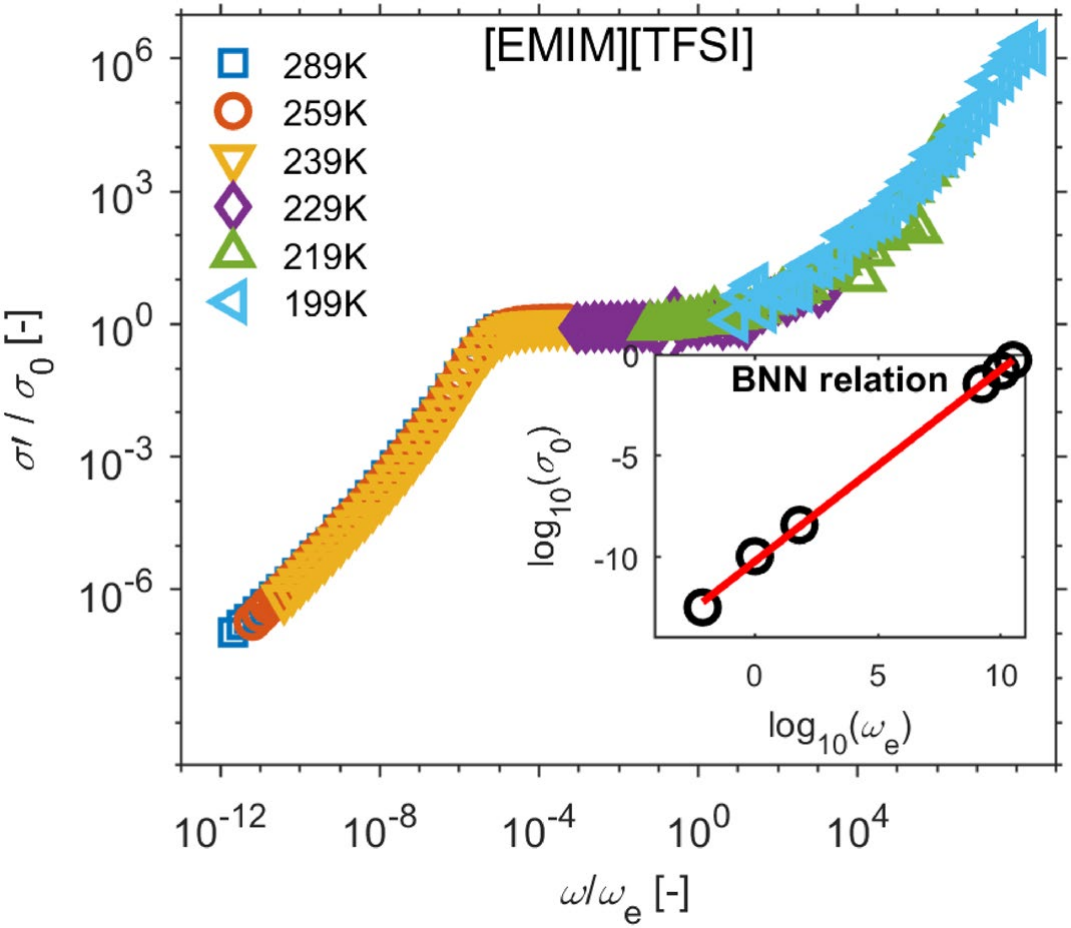
Superimposed conductivity spectra, aka the master curve, of [EMIM] [TFSI] with respect to the charge carrier hopping rate ${\omega }_{e}$ and dc conductivity ${\sigma }_{0}$ for selected temperatures at cooling. The inset represents the validity of the BNN relation for these temperatures.

A number of authors (e.g. Sangoro et al.^39^) demonstrated on imidazolium-based ionic liquids that the dielectric spectra, especially conductivity σ’, could be divided into two spectral regions: (i) the high-frequency part is the charge-dominant regime, while (ii) the low frequency part is dominated by electrode polarization effects. To enlighten these two regions, the spectra in Fig. 2 are normalized with respect to the characteristic frequency at which the real part of conductivity begins to increase with frequency^39^ or corresponds to the frequency of the peak in the imaginary part of modulus (modulus ${M}_{\left(\omega ,T\right)}^{*}=1/ {\varepsilon }_{\left(\omega ,T\right)}^{*}$, not shown here e.g.^40^), and, furthermore, the conductivity spectra in Fig. 2 are normalized with respect to plateaus in σ’. Thus, the normalized curves are shown in Fig. 3 as coinciding dependence, whose low frequency part of the conductivity spectrum σ′ exhibits decrease from the plateau. This is caused by electrode polarization effects which are attributed to the slowing down charge carriers at the electrodes^41–44^.

For many ion-conductive systems, the high-frequency part is well-described by Dyre’s random free energy barrier model^45^ which characterizes the charge transport in disordered ion-conducting solids by the characteristic frequency (i.e. charge carrier hopping rate ${\omega }_{e}=1/{\tau }_{e}$) and the value of the plateau in σ′ (i.e. *dc* conductivity ${\sigma }_{0}$). The model assumes that hopping conduction is the main underlying mechanism in which the charge carriers hop in a random spatially varying energy landscape. Thus, the charge transport is governed by the ability of the charge carriers to overcome the randomly distributed energy barriers^39,43–46^. The characteristic time ${\tau }_{e}$ corresponds to the rate to overcome the highest energy barrier and determines the onset of the *dc* conductivity ${\sigma }_{0}$^43,46^. In other words, this characteristic time separates the dc-regime with diffusive ionic transport from the dispersive regime described by a sub-diffusive ionic motion^46^.

However, a fit of conductivity spectra of the ionic liquid under test exhibits an additional ‘relaxation-like’ process that is assumed^47^ to be connected to structural reorganization of the ionic atmosphere during and after a successful ionic jump. This reorganization causes additional fluctuations in the polarization, thus resulting in a relaxation process with time constant similar to the charge hopping rate.

The complex conductivity is then given as the contribution of a random free energy barrier model (derived in terms of continuous random walk approximation^48^) and an empirical Havriliak-Negami function introduced to conductivity

1 $${\sigma }_{\left(\omega \right)}^{*}={\sigma }_{0} \left(\frac{i\omega {\tau }_{e}}{\mathrm{ln}(1+i\omega {\tau }_{e})} \right)+i\omega {\varepsilon }_{0}\left(\frac{\Delta \varepsilon }{{\left(1+{\left(i\omega {\tau }_{HN}\right)}^{\beta }\right)}^{\gamma }}+{\varepsilon }_{\infty } \right)$$

where $\Delta \varepsilon$ represents the dielectric relaxation strength, ${\varepsilon }_{\infty }$ the relaxed value of ε’ and ${\tau }_{HN}$ Havriliak-Negami relaxation time. The parameters $\beta$ and $\gamma$ describe symmetrical and asymmetrical broadening of the complex dielectric function^47^. Based on the formula, the conductivity spectra σ′ for 199 K, 219 K and 229 K were fitted by using particle swarm optimization^49^.

**Figure 4:**
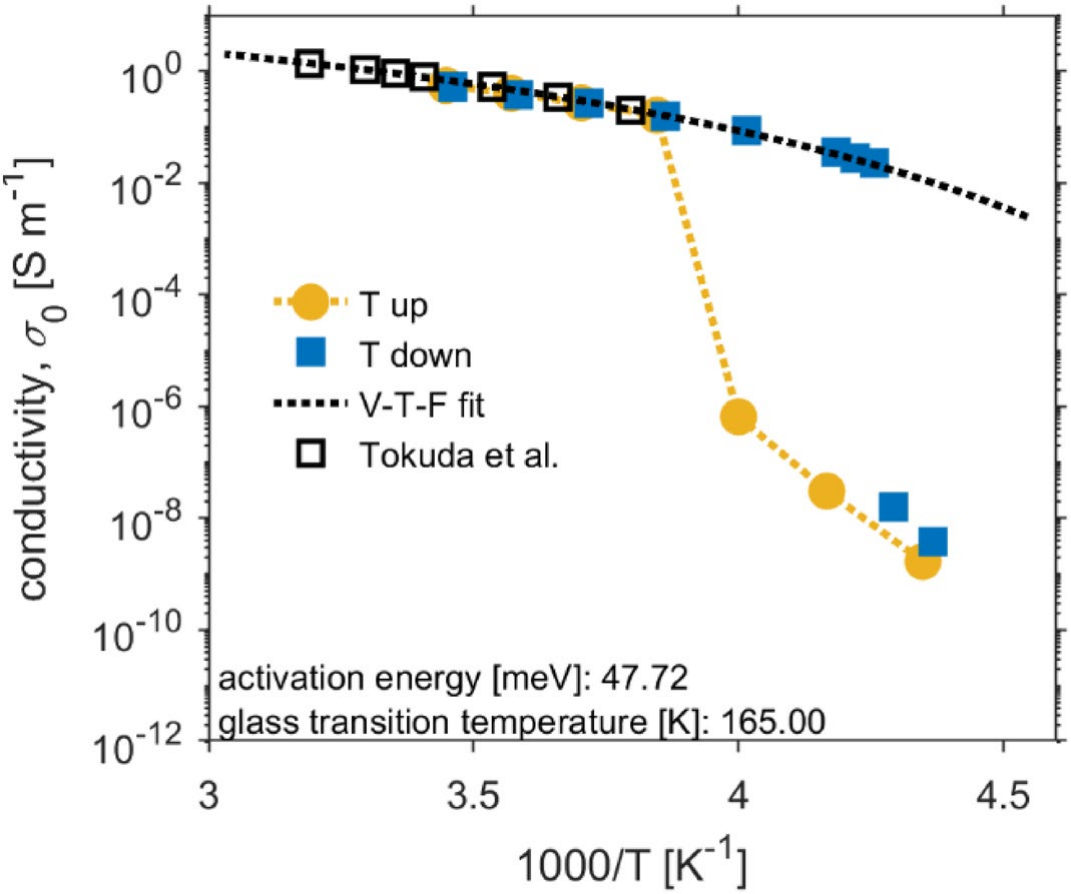
Temperature dependence of ionic conductivity [EMIM][TFSI] (orange circles—heated up; blue squares—cooled down; black dashed line—Vogel–Fulcher–Tammann fit of parameters *E*_a_ = 47.72 meV, *T*_g_ = 165 K, ${\sigma }_{\infty }$ = 58 S/m; black squares—referenced data Tokuda et al.^54^).

The normalized conductivity spectra in Fig. 3, further, demonstrate that the time–temperature superposition principle is observed, just as the Barton–Nakajima–Namikiwa (BNN) relationship ${\sigma }_{0}\sim {\omega }_{e}$ is fulfilled^39,44,46,48^, inset Fig. 3. Thus, ac charge transport and dc charge transport rate share identical thermal activation. Thermal evolution of dc conductivity is shown in the Arrhenius plot (Fig. 4). In a liquid state, this temperature dependence of conductivity is governed by Vogel-Fulcher-Tammann dependence^46,50^. Around the transition between solid and liquid states, a hysteresis loop is apparent, which can be explained as the apparition of supercooled liquid^51^. Several authors dried samples before dielectric measurements^41,44,46,52^ in order not to be contaminated by water molecules from the atmosphere^53^. It needs to be pointed out that our goal is to discuss the influence of thermal treatment on the change of the dc conductivity in the solid polymer electrolyte; therefore, we did not provide such a procedure.

For temperatures above liquid–solid–liquid phase transition, the equation for the temperature dependence of the electrical conductivity is written as follows^50^:

2 $${{\sigma }_{0}}_{\left(T\right)}={\sigma }_{\infty }exp\left(\frac{-{E}_{a}}{{k}_{B}\left(T-{T}_{g}\right)}\right)$$

where *E*_a_ is the activation energy for electrical conduction, ${\sigma }_{\infty }$ is the maximum electrical conductivity, *T*_g_ is glass temperature and *k*_B_ is the Boltzmann constant. Figure 4, furthermore, shows that our measurement is in agreement with experiments already published. The experimental data was fitted by parameters (*E*_a_ = 47.72 meV, *T*_g_ = 165 K, ${\sigma }_{\infty }$ = 58 S/m) that lay within published limits^54,55^.

### Effect of thermal treatment on ac/dc conductivity of SPEs

Solid polymer electrolytes were investigated by the same procedure as the ionic liquid [EMIM][TFSI]. The SPE consisted of three basic components: ionic liquid, polymer matrix and solvent. The frequency-dependent electrical conductivity of neat PVDF rises with the temperature^24,56^. Its real part, σ′, increases with frequency, but also contains an intermediate frequency plateau for all temperatures^56^. The PVDF is the semi-crystalline polymer known for the coexistence between the crystallites and the amorphous state on the microstructure level^57^; thus, several publications reported that neat PVDF exhibits *α*-relaxations (associated with molecular motions within the crystalline fraction of the polymer or in the crystal-amorphous interphase region)^58,59^, *β*-relaxation (associated with cooperative segmental motions within the main chains of the amorphous regions during the glass transition of the polymer)^60^ and Maxwell–Wagner relaxation (an interfacial polarization)^56,59^. Therefore, these relaxation processes may also become particularly relevant in the conductivity spectrum.

Studies on PVDF-based polymer electrolytes without the content of ionic liquids^58,61^ showed that the concentration and type of solvent, together with the drying process, affect the morphology, structure and electrical conductivity. The ionic conductivity is related to the quantity of the trapped solvent (crystallinity of PVDF); thus, the drying process led to a decrease in ionic conductivity^58^. As the solvent polarity decreases, the mobility of polymer chain increases; thus, the interactions between PVDF and solvent are weakened, which favors the formation of the *α*-phase^62^. In contrast, the incorporation of various ionic liquids into the PVDF matrix significantly contributes to the conductivity behavior of the solid polymer electrolyte^1,18,20,25,56,63^. The change of charge carrier movement mechanisms resulted from the increase of ion mobility induced by polymer chain segmental motion and polar phase crystals^18^.

In our case, the mixture (ionic liquid, PVDF and solvent in weight ratio 1:1:3) was thoroughly stirred by a magnetic stirrer at elevated temperature (70 °C) and then a small amount was deposited on a ceramic substrate and kept at a specific temperature (solvent evaporation temperature) for a certain time interval. As the solvent evaporation temperature and time increase, the solvent evaporates faster until it disappears from the SPE mixture (for more details see^38^). It needs to be noted that this limit instance did not happen in our case, since the morphology of the sample would be flat (Fig. 1d).

**Figure 5:**
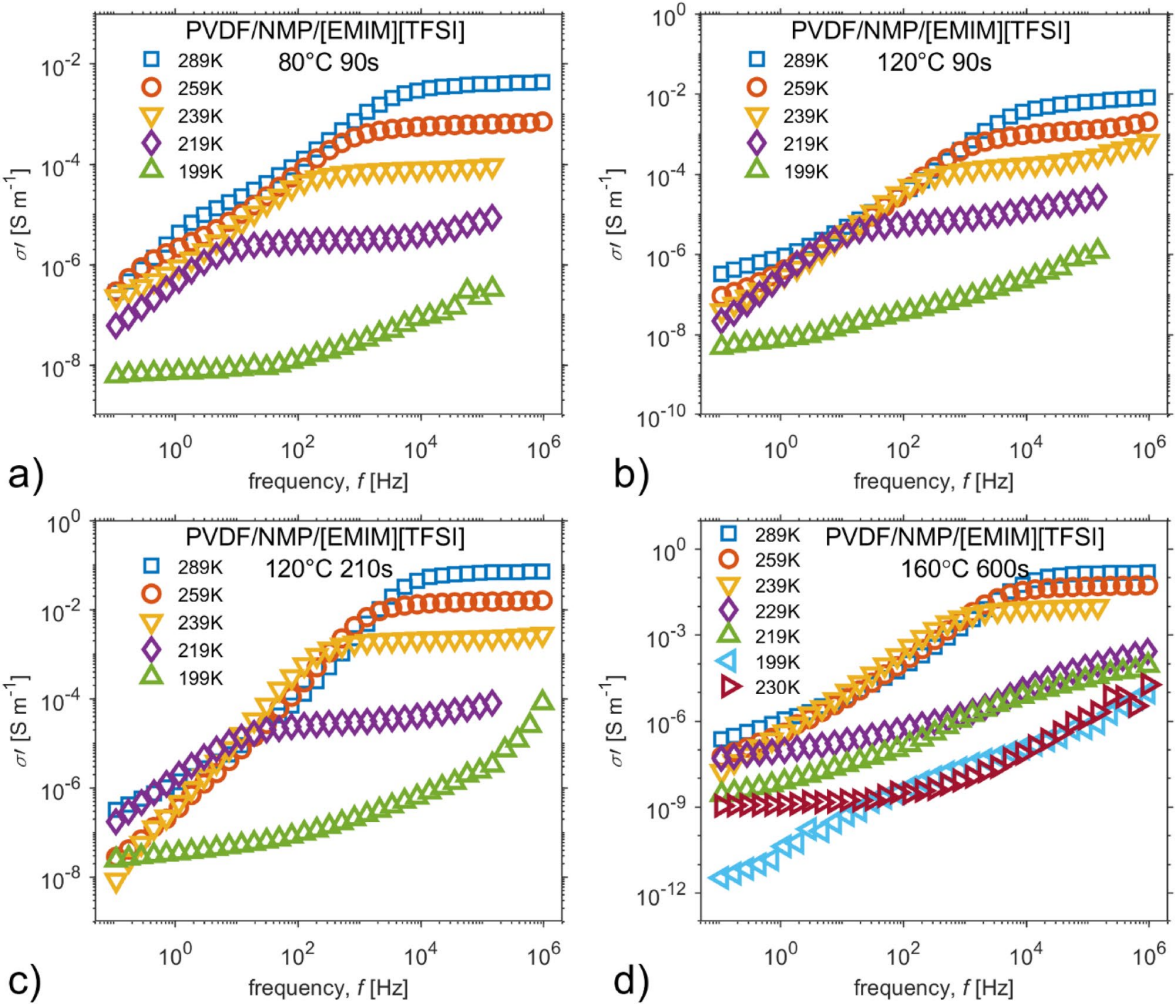
The real part of the conductivity spectra for selected temperatures at cooling; particular SPEs were prepared by the treatment conditions: (**a**) 80 °C for 90 s, (**b**) 120 °C for 90 s, (**c**) 120 °C for 210 s, (**d**) 160 °C for 600 s.

Figure 5 shows how the real parts of conductivity spectra σ′ develop during samples cooling for all four cases of thermal treatment. The ionic conductivity behavior in all SPEs maintains the main characteristics of pure ionic liquid. The σ’ shows in Fig. 5A a relaxation process at a low frequency range for temperatures above 239 K, while Fig. 5d presents a relaxation process around 1 kHz for temperatures below 229 K. The rising intensity of solvent evaporation from the mixture in a preparation process results in sharper transitions of conductivity spectra during sample cooling, occurrence of noticeable relaxation processes and, primarily, an increase in overall conductivity. The ionic conduction occurs predominantly in the amorphous phase or amorphous-crystalline interphase of the polymer. The solvent evaporation rate controls the crystallization of polymer, the matrix crystallinity becomes more amorphous as the rate increases, and, therefore, IL high ionic conductivity is more evident.

**Figure 6:**
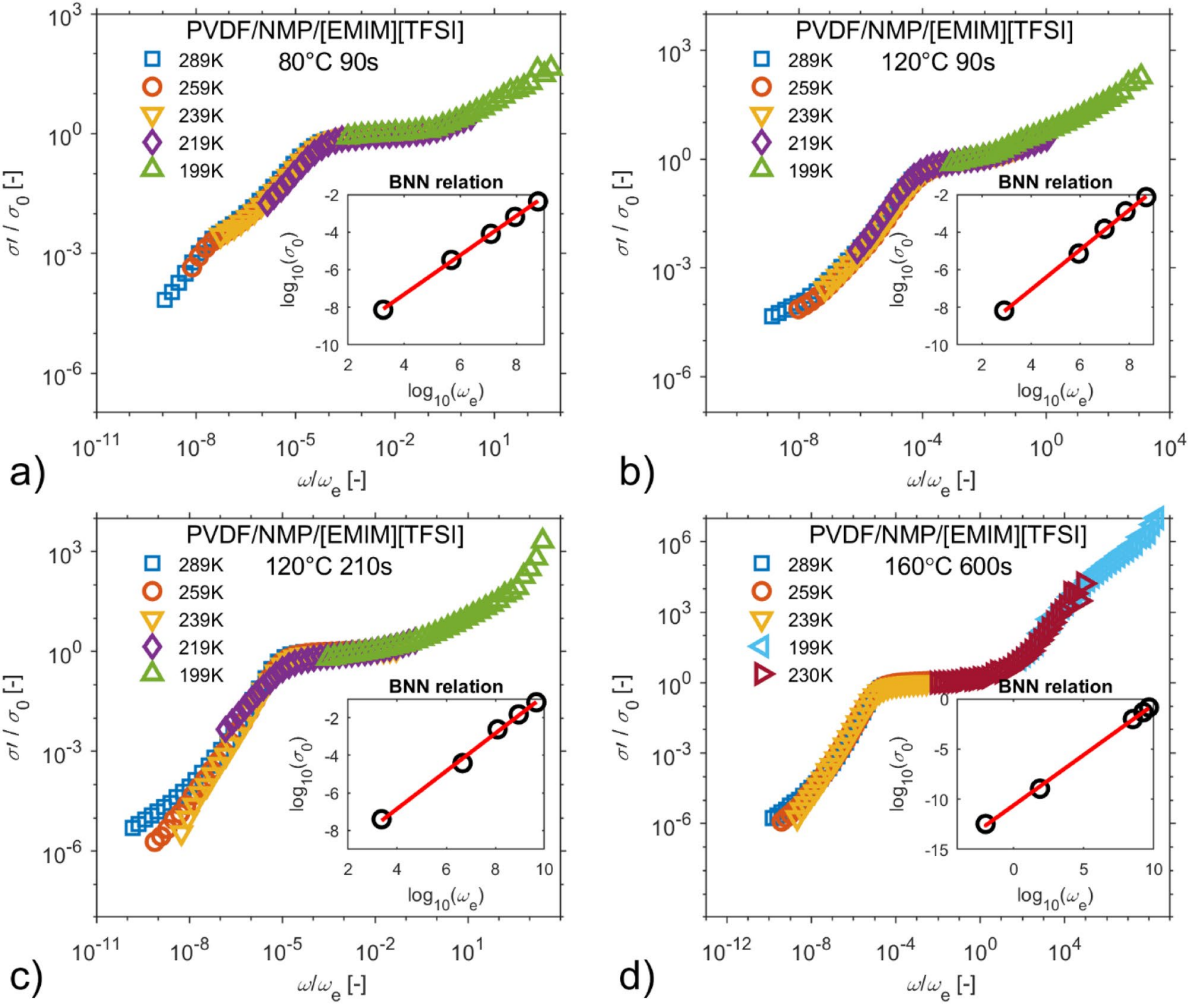
The conductivity master curves with respect to parameters of Dyre’s random free energy barrier model, the inset shows the BNN relations. The particular SPEs were prepared by the following treatment conditions: (**a**) 80 °C for 90 s, (**b**) 120 °C for 90 s, (**c**) 120 °C for 210 s, (**d**) 160 °C for 600 s.

The relaxation process becomes more visible when the time–temperature superposition principle is considered, and normalized conductivity spectra with respect to ${\sigma }_{0}$ and the characteristic frequency ${\omega }_{e}$ are plotted in Fig. 6a–d. In the insets of Fig. 6, the BNN relationships ${\sigma }_{0}\sim {\omega }_{e}$ are shown. Figures 5d and 6d also include real parts of conductivity spectra during sample heating in order to derive a single master curve. The master curves in Fig. 6 exhibit two spectral regions related to different phenomena: the electrode polarization effect and ac conductivity at higher frequencies, which are separated by the plateau of dc conductivity. The shape of the low-frequency region develops as the morphology of SPE changes from small spherical objects to larger ones. The electrode polarization contributes to the dielectric relaxation process at low frequency because the radial and tangential diffusion and accumulation of ions near the electrodes surfaces form an electric double layer^18^. For different amounts of solvent, the initiation of ac conductivity is directly related to the conformational mobility of the system^56^.

**Figure 7:**
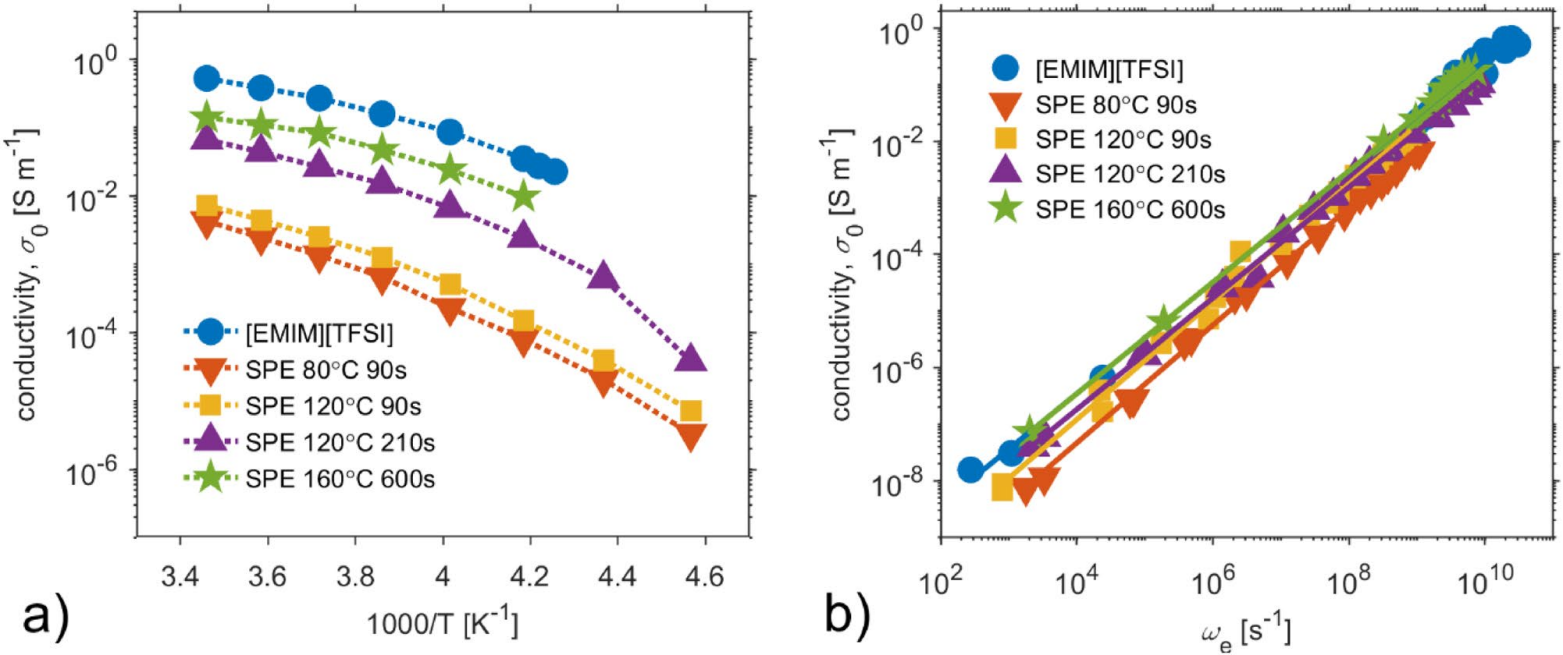
The dc conductivity, ${\sigma }_{0}$, of ionic liquid and PVDF-based SPEs as a function of the different conditions of thermal treatment. (**a**) Arrhenious plot. (**b**) dc conductivity, ${\sigma }_{0}$ as a function of characteristic frequency ${\omega }_{e}$.

Temperature dependences of dc conductivity, shown in the Arrhenius plot (Fig. 7a), exhibit Vogel–Fulcher–Tammann dependence. Figure 7a further shows that dc conductivity increases with the intensity of thermal treatment and is connected to solvent amount in mixtures. All SPEs exhibit a phase transition hysteresis loop, caused by the ionic liquid used. With higher treatment temperature, the ionic conductivity of ionic liquid in SPEs prevails, since liquid–solid-liquid phase transition hysteresis loop of ionic liquid becomes more significant.

Figure 7b summarizes the insets of Fig. 6a–d and Fig. 3. The dependences of dc conductivity ${\sigma }_{0}$ on characteristic frequency ${\omega }_{e}$ of ionic liquid and all SPEs imply that the glassy dynamics enhances charge transport in ionic liquid. The overlap of characteristic linear relationship between ${\sigma }_{0}$ and ${\omega }_{e}$ (BNN) is better as the SPE conductivity approaches to conductivity of the ionic liquid.

### Effect of thermal treatment on current fluctuations in SPEs

Experimental noise measurements will inherently be somewhat uncertain. One of the challenges of experimental work is to minimize the uncertainty, especially for high impedance samples such as polymer electrolytes and/or liquids with ion containments. In both cases, the internal impedance is dynamically changed with respect to the electrical excitation state and the dielectric relaxation state in the time domain.

The investigation of current fluctuations was carried out on samples which were prepared on a golden IDE (four different preparation conditions) and a platinum IDE (two different preparation condition). The goal was to find the impact of SPE conductivity or electrode material on current noise spectral density. The excitation of the samples was done by low-noise voltage sources, i.e. NiMH batteries (1.0 V, 1.4 V) and NiCd battery 3.1 V.

**Figure 8:**
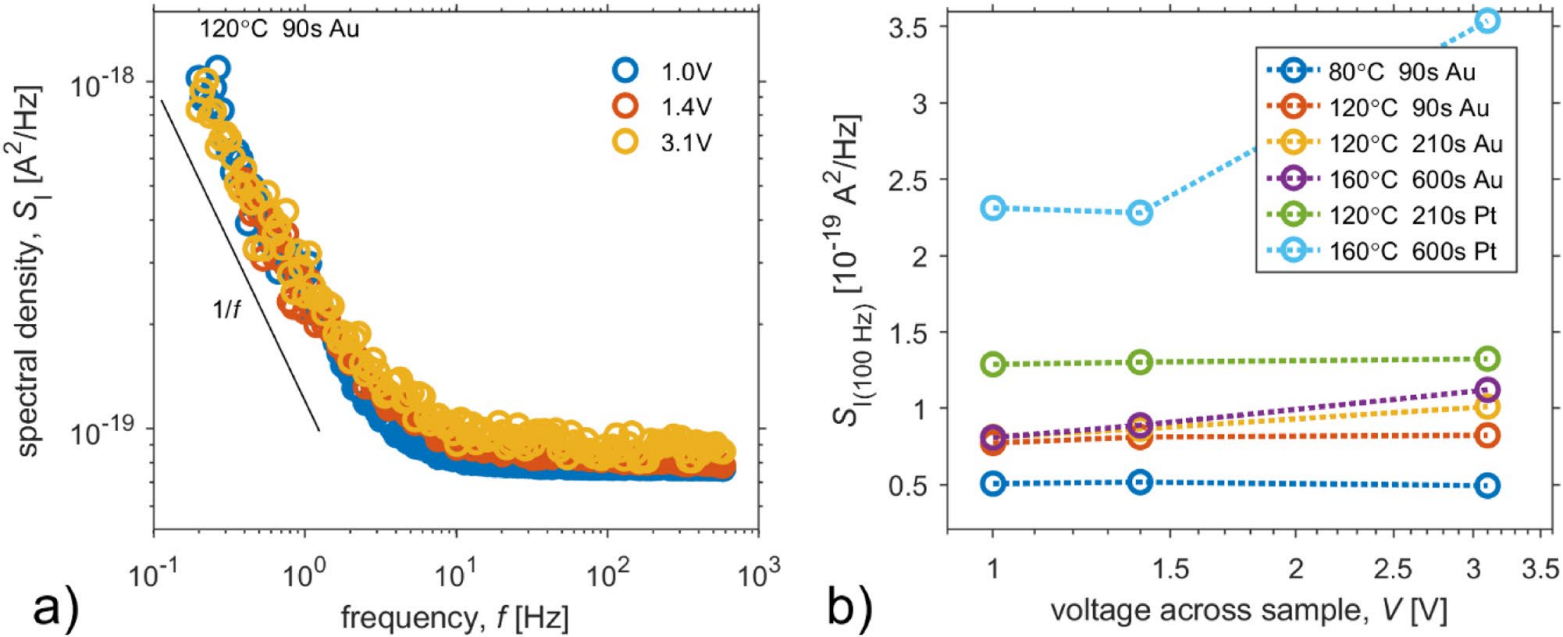
(**a**) Spectral density of current fluctuations at voltages 1.0 V, 1.4 V, 3.1 V. Measurements were provided on a sample prepared on a golden IDE under the condition of 120 °C for 90 s. (**b**) Spectral density of current fluctuations at frequency 100 Hz as a function of applied voltage for samples prepared under a range of preparation conditions on a golden or platinum IDE.

Figure 8a show typical spectral density of current fluctuations. All samples produce flicker noise and white noise. It needs to be pointed out that in our experiments, the contribution of flicker noise shows less significant dependence on the type of the SPE unlike the white noise component. To illustrate the dependence of the white noise component, the values of current noise spectral density at the frequency of 100 Hz corresponding to the spectral plateau were plotted to Fig. 8b, where two noise sources can be distinguished: thermal noise and shot noise. Both of them depends on charge transfer resistance of the electrode/electrolyte interface^33^ and bulk resistance. When the white noise component shows no current dependency, the thermal noise, originating from all dissipative components, prevails. When the white noise component shows a dependency on current via sample, the shot noise, depending on the ratio of the resistances^64^, prevails. Thus, Fig. 8b shows that decrease in bulk resistance (i.e. increase in electrolyte conductivity) or charge transfer resistance leads to increase in the shot noise component, as well as the DC current via a sample. The change of electrode material results in a more significant change than the modification of the preparation condition of the solid polymer electrolyte. Thus contact noise, connected to current crowding^65,66^ and the charge transfer resistance, contributes significantly more than the current fluctuations generated in SPEs. To minimize the effect of the contact noise, the electrode geometry should be modified to have the optimal perimeter-to-area ratio of electrode/electrolyte interface.

The flicker noise (1/f a-like- noise) could be explained by strong contribution from contacts (i.e. 1/*f* contact noise^65^), while Hassibi et al.^33^ described 1/f noise at electrode/electrolyte interference as result of diffusion-dominant electrode, where a low interfacial electrical field only leads to diffusion movements of little drift, i.e. diffusion of current carriers (ions).

## Summary

Using broadband dielectric spectroscopy, the ac/dc conductivities of the ionic liquid [EMIM][TFSI] and SPEs were investigated over a broad range of temperatures. The frequency-dependent conductivity data were analysed using the free energy random barrier model and established a time–temperature superposition.

The conductivity spectra of the ionic liquid are given as the contribution of a random free energy barrier model and an additional ‘relaxation-like’ process which is presumably connected to the structural reorganization of the ionic atmosphere during and after a successful ionic jump.

In SPE samples of the higher solvent evaporation rate, the real parts of conductivity spectra exhibit a sharper transition during sample cooling and an increase in overall conductivity, which is implied by a growing fraction of an amorphous phase in the polymer matrix in which the ionic liquid is immobilized. The conductivity master curves illustrate that the changing SPEs morphology is reflected in the low frequency regions governed by the electrode polarization effect. The initiation of ac conductivity is directly related to the conformational mobility of the system. The Barton–Nakajima–Namikiwa relation was valid for all samples; thus, ac charge transport and dc charge transport have identical thermal activation barriers. The overlap of characteristic linear relationship between ${\sigma }_{0}$ and ${\omega }_{e}$ (BNN) is better as the SPE conductivity approaches to conductivity of the ionic liquid; therefore, the ionic conductivity behavior of ionic liquid is assumed to prevail in all four types of the prepared SPEs. Temperature dependences of dc conductivity exhibit Vogel–Fulcher–Tammann dependence. The dc conductivity of SPEs increases with the intensity of thermal treatment and is connected to solvent evaporation.

Noise measurement reveals that flicker noise, thermal noise, shot noise and contact noise are major sources of current fluctuation in all samples. Thermal noise and shot noise are related to charge transfer resistance of the electrode/electrolyte interface and bulk resistance. The increase of electrolyte conductivity causes a decrease in bulk resistance and partially a decrease in charge transfer resistance, while also resulting in an increase in the shot noise. However, the change of electrode material results in a more significant change of spectral density of current fluctuations than the modification of the preparation condition of the solid polymer electrolyte. The contact noise, connected to electrical current crowding and interphase resistance, contributes to current fluctuations of white noise components and also could contribute to flicker noise. The electrochemical processes, taking place at the electrode/electrolyte interface, lead to a significantly higher response of current fluctuations in comparison with processes that participate in the charge transport in the electrolyte. This is in accordance with the generally accepted knowledge that the electrode/electrolyte interface plays a key role in charge transfer through every electrochemical device. Thus, the flicker noise could also indicate diffusion of current carriers (ions).

## Methods

### Sample preparation

The electrode platform is based on a ceramic substrate, the thickness of which was 0.7 mm, with gold interdigital (comb-like) electrodes, which was manufactured by lift-off technology. The thickness of the gold electrodes was in the order of hundreds of nanometers and the finger/gap width was equaled 25/25 µm, respectively. The SPE consisted of three basic components: (i) ionic liquid 1-ethyl-3-methylimidazolium bis(trifluoromethylsulfonyl)imide ([EMIM][TFSI]) (ii) poly-(vinylidene fluoride) (PVDF), (iii) *N*-methyl-pyrrolidone (NMP) as a solvent. All the components were mixed together (1:1:3 wt., respectively) and the mixture was thoroughly stirred by a magnetic stirrer at elevated temperature (70 °C). Subsequently, the amount of 1.5 mg of the mixture was deposited by drop coating onto the ceramic substrate in order to prepare the SPE layer. After the deposition, the alumina substrate with the SPE layer was placed on a hot plate where the sample was kept at an appropriate temperature for a specific time in order to achieve different microstructure and porosity of the SPE layer, i.e., to achieve different crystalline forms of the PVDF. Four different conditions for crystallization of PVDF in NMP were used: (a) 80 °C for 1.5 min, (b) 120 °C 1.5 min, (c) 120 °C for 3.5 min and (d) 160 °C for 10 min. These conditions were chosen empirically because of good SPE layer adhesion to the ceramic substrate, which is important for subsequent electrical characterization.

### Conductivity measurements by dielectric spectroscopy

The conductivity of the prepared samples was investigated on the basis of wide broadband dielectric spectroscopy. The electrical impedance spectra of every sample were measured by an Alpha-A Analyzer (Novocontrol Technologies) in the wide frequency range from 10 mHz up to 1 MHz and at temperatures from 289 K down to 199 K and up to 290 K in the vacuum of 0.1 Pa. These measurements were carried out in helium cryostat (Janis Research Company, helium pump—SHI Cryogenics, temperature controller—LakeShore, vacuum pump—Agilent Technologies).

Since the samples were prepared as a thin layer on an alumina substrate with a golden interdigital electrode (IDE), the complex dielectric function ${\varepsilon }^{*}$, as well as complex electric conductivity ${\sigma }^{*}$, cannot be directly calculated from the electrical impedance spectra, which also contain information about the dielectric function of substrate ${\varepsilon }_{sub}^{*}$ and vacuum capacity ${C}_{0}^{*}$^67^. These two characteristics were obtained by measurements of the blank IDE under conditions specified above and measurements of an IDE with deposited glycerol in the temperature range between 290 and 310 K at atmospheric pressure. Thus, the complex dielectric function ${\varepsilon }^{*}$ was estimated on the basis of IDE experimental data and the calculated complex dielectric function of substrate ${\varepsilon }_{sub}^{*}$ and the calculated complex vacuum capacitance ${C}_{0}^{*}$^67^. This procedure was successfully verified on the measurement of the ionic liquid [EMIM][TFSI], where the estimated complex dielectric was compared with measurement carried out in parallel-plate cell for liquids (BDS1308 + Alpha Active Cell—Novocontrol Technologies) at room temperature. The complex electrical conductivity ${\sigma }^{*}$ at a certain angular frequency $\omega$ and a certain temperature *T* is then given by the complex dielectric function as

3 $${\sigma }_{\left(\omega ,T\right)}^{*}=i{\varepsilon }_{0}\omega {\varepsilon }_{\left(\omega ,T\right)}^{*}$$

where *i* is an imaginary unit and ${\varepsilon }_{0}$ vacuum permittivity. The dc conductivity ${\sigma }_{0}$ is estimated from plateaus in spectra of a real part of complex conductivity ${\sigma }_{\left(\omega ,T\right)}^{*}$.

### Noise measurements

Experimental noise measurements will inherently be somewhat uncertain. One of the challenges of experimental work is to minimize the uncertainty especially for high impedance samples such as polymer electrolytes and/or liquids with ion containments. In both cases, the internal impedance is dynamically changed with respect to the electrical excitation state and the dielectric relaxation state in the time domain. The samples of solid polymer electrolytes pointed out strong inertial properties, requiring meticulous care immediately before measurement. There is meant the stabilization of the current response after a stepwise application of voltage, the inclination of the sample and consistent electrical shielding. The internal impedance is in the order of hundreds of MOhms, and even here, the concept of the measuring circuit must be carefully chosen. The excitation of the samples was performed with NiMH batteries (1.04 V) with a sufficient time interval from charging, and a resistor as a current transducer was included in series with the sample. The low noise current preamplifier (Signal Recovery SR570) was used and a transimpedance gain along with input circuits balancing was performed by the transimpedance resistance selection. The low-pass filter was parasitically formed (cut-off frequency of about 25 Hz), but the background noise was reduced and the response of the samples to voltage excitation appears to be relevant. It should be noted here that DC excitation causes saturation of the DC couplet current preamplifier input and must be compensated by the amplifier DC setting. The key is the choice of the sensing resistance with respect to the noise figure of the connection as a whole. Since the parasitic noise contributions in the equivalent circuit are uncorrelated, the resulting noise background is equal to the square root of the sum of the squares of the individual contributions. In our case, however, parasitic voltage and current contributions are applied, and, therefore, the characteristic noise resistance U_n_/I_n_ (referred to amplifier input) was determined. The same value was then set as the sum of *R*_sample_ + *R*_sense_, while ensuring balance and matched conditions. In this sensing configuration, a number of measurements were performed; it was shown that the samples produce significant flicker noise and the main limiting factor is a parasitically formed low-pass filter in the input circuits. Fortunately, this type of noise has dominant components in the very low frequency range and relevant conclusions could be drawn.

## Data Availability

The datasets measured and analyzed during the current study are available from the corresponding author on reasonable request.
